# *Escherichia coli* and Community-acquired Gastroenteritis, Melbourne, Australia

**DOI:** 10.3201/eid1010.031086

**Published:** 2004-10

**Authors:** Roy M. Robins-Browne, Anne-Marie Bordun, Marija Tauschek, Vicki R. Bennett-Wood, Jacinta Russell, Frances Oppedisano, Nicole A. Lister, Karl A. Bettelheim, Christopher K. Fairley, Martha I. Sinclair, Margaret E. Hellard

**Affiliations:** * University of Melbourne, Melbourne, Victoria, Australia;; † Murdoch Children's Research Institute, Melbourne, Victoria, Australia;; ‡ Monash University, Melbourne, Victoria, Australia

**Keywords:** Diarrhea, enteropathogenic E. coli, Escherichia coli infections, gastroenteritis, research

## Abstract

As part of a study to determine the effects of water filtration on the incidence of community-acquired gastroenteritis in Melbourne, Australia, we examined fecal samples from patients with gastroenteritis and asymptomatic persons for diarrheagenic strains of *Escherichia coli*. Atypical strains of enteropathogenic *E. coli* (EPEC) were the most frequently identified pathogens of all bacterial, viral, and parasitic agents in patients with gastroenteritis. Moreover, atypical EPEC were more common in patients with gastroenteritis (89 [12.8%] of 696) than in asymptomatic persons (11 [2.3%] of 489, p < 0.0001). Twenty-two random isolates of atypical EPEC that were characterized further showed marked heterogeneity in terms of serotype, genetic subtype, and carriage of virulence-associated determinants. Apart from the surface protein, intimin, no virulence determinant or phenotype was uniformly present in atypical EPEC strains. This study shows that atypical EPEC are an important cause of gastroenteritis in Melbourne.

## Abstract 2

Atypical strains of enteropathogenic *E. coli* are a leading cause of gastroenteritis in Melbourne.

Strains of *Escherichia coli* that cause diarrhea are classified into pathotypes (or virotypes) according to their specific virulence determinants (*1*). These virulence determinants give each pathotype the capacity to cause a clinical syndrome with distinctive epidemiologic and pathologic characteristics. For example, enterohemorrhagic *E. coli* (EHEC) may cause hemorrhagic colitis and the hemolytic uremic syndrome because of their production of Shiga toxins, whereas enteroaggregative *E. coli* (EAEC) are associated with persistent diarrhea in children in less-developed countries (*1*). Enteropathogenic *E. coli* (EPEC) share several key virulence determinants with the most common varieties of EHEC, but lack Shiga toxins, and cause nonspecific diarrhea in infants in less-developed countries (*2**,**3*). EPEC also differ from EHEC in that they typically carry an EPEC adherence factor plasmid (EAF). This plasmid encodes both bundle-forming pili (Bfp) that promote bacterial adherence to mammalian cells and are required for virulence (*4*) and a transcriptional activator, known as Per, that upregulates genes, such as *eae*, within a pathogenicity island termed the locus for enterocyte effacement (LEE) (*5*). LEE is required to produce attaching-effacing lesions, which are characteristic of EPEC-induced pathology. A subset of EPEC, known as atypical EPEC, does not carry EAF and hence does not produce Bfp (*3*). The role of EPEC in disease is uncertain.

The principal reservoir of EHEC is food animals, in particular, cattle, which harbor these bacteria in the distal intestinal tract and from which bacteria can spread to humans through fecally contaminated food or water (*1*). Although the other pathotypes of diarrheagenic *E. coli* generally do not originate in animals, they may also spread to humans through food or water contaminated with excrement. Recently, we conducted a study to determine if the water supply of Melbourne, Australia's second largest city with >3 million inhabitants, is a source of intestinal pathogens that are responsible for community-acquired gastroenteritis. Among the pathogens that were sought were diarrheagenic *E. coli*, including atypical EPEC, which emerged as the predominant cause of gastroenteritis in this community.

## Materials and Methods

The design of the Water Quality Study (WQS), which was conducted from September 1997 to February 1999, has been reported previously (*6*). Briefly, 600 Melbourne families, with at least two children 1–15 years of age, were enrolled in the study. Each family was allocated at random to receive a real or sham water treatment unit, which was installed in the kitchen of their home and supplied water through a separate faucet. Family members, comprising 2,811 persons, were followed for 15 months (68 weeks). Each participating household had a nominated member who completed a weekly questionnaire regarding the presence, duration, and severity of gastrointestinal symptoms. The primary endpoint of the study was highly credible gastroenteritis, which was defined as exhibiting any of the following symptoms in a 24-hour period: two or more loose stools, two or more episodes of vomiting, one loose stool together with abdominal pain or nausea or vomiting, or one episode of vomiting with abdominal pain or nausea. Cases of highly credible gastroenteritis were deemed to be distinct if the participant was symptom-free for at least 6 days.

### Sample Collection and Processing

Participants in the study were asked to collect fecal specimens during episodes of gastroenteritis. A total of 795 specimens collected during 2,669 reported episodes of gastroenteritis were examined for rotavirus, adenovirus, Norwalk-like viruses, *Giardia* spp., and *Cryptosporidium* spp. and were cultured for *Salmonella* spp., *Shigella* spp., *Campylobacter* spp., *Vibrio* spp., *Yersinia* spp., *Aeromonas* spp., *Plesiomonas* spp., and *Clostridium difficile*, as described previously (*6**,**7*). Baseline frequencies of these pathogens in the study population were determined during the 4-month period, May through August 1997, immediately preceding the WQS. Frequencies were examined by investigating 1,091 fecal specimens from a convenience sample of participants. Participants who provided a baseline specimen were similar to those who did not provide a specimen in age, sex, and family background.

### Examination of Feces for *E. coli*

Sufficient funds were available to investigate 1,250 samples for diarrheagenic *E. coli*. Of these samples, 500 were randomly selected from 1,091 fecal samples obtained from healthy persons in the baseline study, and 750 samples were randomly selected from the 795 samples obtained from participants with highly credible gastroenteritis in the WQS.

Bacteria were isolated from fecal samples by direct plating on MacConkey agar (Oxoid Ltd., Basingstoke, UK). After overnight incubation at 37°C, a sterile cotton swab was used to transfer the entire growth from each plate into Luria broth containing 30% (vol/vol) glycerol, which was then frozen at –70°C until required. Diarrheagenic strains of *E. coli* were identified by polymerase chain reaction (PCR) and confirmed by Southern hybridization. Template DNA for use in PCR was prepared from bacteria grown in 2.5 mL of MacConkey broth that contained a loopful of stored frozen culture and was incubated with shaking at 37°C overnight. One milliliter of this culture was centrifuged to pellet the bacteria, the supernatant was removed, and then the pellet was washed in 1 mL of phosphate buffer, resuspended in 200 µL sterile distilled water, and heated for 10 min at 100°C. Samples were then placed on ice for 5 min and recentrifuged at 16,000 x *g*. Aliquots of the supernatant were pipetted into sterile tubes and stored for <1 week at –20°C before use.

PCR amplifications were performed in a Gene Amp PCR System 9700 thermal cycler (Applied Biosystems, Foster City, CA) with AmpliTaq Gold polymerase (Applied Biosystems) and the primers listed in Table 1 in a reaction volume of 20 µL (for single reactions) or 50 µL (for multiplex PCR). The genes identified by these primers and their association with each pathotype of diarrheagenic *E. coli* are listed in Tables 1 and 2. PCR for the *lacZ* gene, which is found in almost all wild-type strains of *E. coli*, was included as a control to ensure that negative PCR assays were not a result of the absence of viable bacteria in the sample or the presence of inhibitors in the reaction mixture. Samples that were negative in the PCR for *lacZ* (3.6% of all those examined) were excluded from further analysis. At the conclusion of the PCR, 10 mL of the reaction mixture underwent electrophoresis on 2.5% 96-well format agarose gels (Electro-fast, ABgene, Epsom, UK). Gels were stained with ethidium bromide, visualized on a UV transilluminator, and photographed. A portion of the PCR product was retained for Southern blotting, which was performed by using capillary transfer of separated DNA fragments onto positively charged nylon membranes (Roche Diagnostics Ltd., Lewes, UK). Digoxigenin-labeled DNA probes were prepared by PCR (Roche Diagnostics) from the control strains (Table 2) by using the PCR primers listed in Table 1. The integrity of the probes was determined by nucleotide sequencing. Probes were hybridized overnight under conditions of high stringency at 65°C and detected by using chemiluminescence as recommended by the manufacturer. Probe-positive bacteria were assigned to a pathotype according the criteria in Table 2. Equivocal or ambiguous assays were repeated, and if results were still unclear, these specimens were excluded from further analysis.

**Table 1:** Characteristics of the polymerase chain reaction (PCR) primers used to detect pathogenic *Escherichia coli* in mixed culture

Target gene or virulence factor	Primer^a^	Primer sequence (5´ to 3´)	PCR program (30 cycles)^b^	Product size (bp)	Reference
*eae*	1^1^ 2^1^	GACCCGGCACAAGCATAAGC CCACCTGCAGCAACAAGAGG	95°C, 30 s; 54°C, 90 s; 72°C, 90 s	384	(*8*)
*ehxA*	1^1^ 2^1^	GCATCATCAAGCGTACGTTCC AATGAGCCAAGCTGGTTAAGCT		534	(*8*)
*stx1*	1^2^ 2^2^	ATAAATCGCCATTCGTTGACTAC AGAACGCCCACTGAGATCATC	95°C, 30 s; 52°C, 60 s; 72°C, 60 s^c^	180	(*8*)
*stx2*	1^2^ 2^2^	GGCACTGTCTGAAACTGCTCC TCGCCAGTTATCTGACATTCTG		255	(*8*)
*ltA*	1^3^ 2^3^	GGCGACAGATTATACCGTGC CCGAATTCTGTTATATATGTC	94°C, 60 s; 50°C, 60 s; 72°C, 120 s^c^	696	(*9*)
*st1A*	1^3^ 2^3^	TCTGTATTATCTTTCCCCTC ATAACATCCAGCACAGGC		186	(*9*)
*ipaC*	1 2	CAGCAGATTGCAGCGCATAT CAAGAGCAGATGCATAACGC	94°C, 30 s; 59°C, 90 s; 72°C, 90 s	811	This study
*bfpA*	1 2	ATTGAATCTGCAATGGTGC ATAGCAGTCGATTTAGCAGCC	95°C, 40 s; 55°C, 40 s; 72°C, 40 s	461	This study
pCVD432	1 2	CTGGCGAAAGACTGTATCAT CAATGTATAGAAATCCGCTGTT	94°C, 40 s; 53°C, 60 s; 72°C, 60 s	630	(*10*)
*aggA*	1 2	ATGCATTACTTTGGGTTTAG TCAACCTTGACACTTGCC	94°C, 60 s; 50°C, 60 s; 72°C, 120 s^c^	414	This study
*lacZ*	1 2	TGATTGAAGCAGAAGCCTGC CGCCAATCCACATCTGTGAA	94°C, 30 s; 59°C, 90 s; 72°C, 90 s	1,350	This study

^a^Primers with the same superscript were used in duplex PCRs. ^b^Before the first cycle, the sample was denatured for 10 min at 95°C; after the last cycle, the sample was extended for 8 min at 72°C. ^c^35 cycles.

**Table 2:** Classification of pathogenic *Escherichia coli* according to amplicon(s) generated by polymerase chain reaction for virulence-associated determinants

Interpretation^b^	Gene or virulence-associated determinant^a^											
	pCVD432	*aggA*	*bfpA*	*eae*	*exhA*	*ipaC*	*stIA*	*ltA*	*stx1*	*stx2*	Control strain	Reference
EAEC^c^	+	+	–	–	–	–	–	–	–	–	O42	(*11*)
Typical EPEC	–	–	+	+	–	–	–	–	–	–	E2348/69	(*12*)
Atypical EPEC	–	–	–	+	–	–	–	–	–	–	E128012	(*12*)
EIEC	–	–	–	–	–	+	–	–	–	–	223/83	(*13*)
ETEC^c^	–	–	–	–	–	–	+	+	–	–	H10407	(*14*)
EHEC^d^	–	–	–	+	+	–	–	–	+	+	EDL933	(*15*)
STEC, not EHEC^e^	–	–	–	–	–	–	–	–	+	+		

^a^Factors specified by virulence genes: *aggA*, aggregative fimbria, AAF/I; *bfpA*, bundle-forming pilus; *eae*, intimin; *ipaC*, invasion plasmid antigen; *stIA*, heat-stable enterotoxin; *ltA*, heat-labile enterotoxin; *stx*, Shiga toxin. ^b^EAEC, enteroaggregative *E. coli*; EPEC, enteropathogenic *E. coli*; EIEC, enteroinvasive *E. coli*; ETEC, enterotoxigenic *E. coli*; STEC, Shiga toxin–producing *E. coli*; EHEC, enterohemorrhagic *E. coli*. ^c^Either *stlA* or *ltA* positive. ^d^Either *eae* or *ehxA* and *stx1* or *stx2* positive. ^e^Either *stx1* or *stx2* positive.

### Characterization of Isolates

Twenty-two EPEC isolates (11 from healthy persons and 11 from persons with diarrhea) were isolated in pure culture. Representative colonies of each were then serotyped with hyperimmune rabbit antisera (*16*). These strains were also subjected to PCR with the primers and conditions listed in Table 3 to determine intimin subtype and to investigate the presence of selected virulence-associated genes. The same 22 strains were also examined for their ability to adhere to and invade HEp-2 epithelial cells.

**Table 3:** Characteristics of the polymerase chain reaction (PCR) primers used in this study for strain characterization

Primer	Primer sequence (5´ to 3´)	Target	PCR program (30 cycles)^a^	Product size (bp)	Reference
P1^b^	CTGAACGGCGATTACGCGAA				
P2	CCAGACGATACGATCCAG	*eae* ^c^	94°C, 30 s; 53°C, 30 s; 72°C, 60 s	917	(*17*)
P3	CTGGAGTTGTCGATGTT	*eae-α*	94°C, 30 s; 53°C, 30 s; 72°C, 120 s	1,648	(*17*)
P4	GTAATTGTGGCACTCC	*eae-β*	94°C, 30 s; 53°C, 30 s; 72°C, 120 s	1,926	(*17*)
P5	GCCTCTGACATTGTTAC	*eae-γ*	94°C, 30 s; 53°C, 30 s; 72°C, 120 s	1,770	(*17*)
ecsD-lower	TATTTTCAAAAAGAATGATGTC	*eae*	94°C, 30 s; 56°C, 60 s; 72°C, 150 s	»2,990^d^	(*18*)
SK1^e^	CCCGAATTCGGCACAAGCATAAGC				
LP5	AGCTCACTCGTAGATGACGGCAAGCG	*eae-ε*	94°C, 30 s; 55°C, 60 s; 72°C, 120 s	2,608	(*18*)
LP6B	TAGTTGTACTCCCCTTATCC	*eae-ζ*	94°C, 30 s; 53°C, 60 s; 72°C, 150 s	2,430	(*18*)
LP7	TTTATCCTGCTCCGTTTGCT	*eae-ι*	94°C, 30 s; 52°C, 60 s; 72°C, 150 s	2,685	(*18*)
LP8	TAGATGACGGTAAGCGAC	*eae-η*	94°C, 30 s; 52°C, 60 s; 72°C, 150 s	2,590	(*18*)
LP10	GGCATTGTTATCTGTTGTCT	*eae-κ*	94°C, 30 s; 52°C, 60 s; 72°C, 150 s	2,769	(*18*)
LP11B	GTTGATAACTCCTGATATTTTA	*eae-θ*	94°C, 30 s; 50°C, 60 s; 72°C, 150 s	2,686	(*18*)
LPFDF LPFDR	GAACTGTAGATGGGTAC AGCAGGCATAACGCAAG	*lpfD*	94°C, 60 s; 48°C, 50 s; 72°C, 60 s	798	(*19*)
Donne-280 Donne-281	CGGAACAGTAGGTTCACCTTC AGTGCCCGTGTTCTTGAACTG	*efa1*	94°C, 30 s; 50°C, 30 s; 72°C, 120 s	2,226	(*20*)
EASTOS1 EASTOS2	GCCATCAACACAGTATATCCG CGCGAGTGACGGCTTTGTAG	*astA*	94°C, 30 s; 50°C, 60 s; 72°C, 90 s	109	(*10*)
AggRks1 AggRkas2	GTATACACAAAAGAAGGAAGC ACAGAATCGTCAGCATCAGC	*aggR*	94°C, 30 s; 50°C, 60 s; 72°C, 45 s^f^	254	(*21*)

^a^Before the first cycle, the sample was denatured for 10 min at 95°C; after the last cycle, the sample was extended for 7 min at 72°C. ^b^Primer P1 was used as forward primer in all PCR reactions in combination with P2, P3, P4, and escD-lower. ^c^Conserved region of *eae*. ^d^Amplicon sequenced. ^e^Primer SK1 was used as forward primer in all PCR reactions in combination with LP5, LP6B, LP7, LP8, LP10, and LP11B. ^f^35 cycles.

### Bacterial Adhesion and Invasion of HEp-2 Cells

The Center for Vaccine Development (University of Maryland, Baltimore, MD) method was used to determine the pattern of bacterial adherence to HEp-2 epithelial cells (*12*). Bacterial strains were designated nonadherent if <10 of 200 HEp-2 cells had five or more bacteria attached. The fluorescent actin staining (FAS) assay, which correlates with the ability of *E. coli* to produce attaching-effacing lesions in the intestine, was performed by using a 6-h incubation period (*22*). At the completion of the assay, cells were examined by fluorescence and phase-contrast microscopy to confirm that fluorescent areas corresponded to attached bacteria. Bacterial strains that gave equivocal or negative results in the FAS assay were investigated for DNA corresponding to regions of the LEE, other than *eae*, by colony blotting using the LEE-A, LEE-B, and LEE-D DNA probes and hybridization conditions described previously (*23*). Quantitative assessment of the ability of *E. coli* to invade HEp-2 cells was performed with the gentamicin-protection assay (*24*). Results were expressed as the number of bacteria recovered from HEp-2 cells after treatment with gentamicin as a percentage of the total number of cell-associated bacteria (*24*).

Statistical analysis was performed with InStat version 3.0 (GraphPad Software Inc., San Diego, CA). A p value < 0.05 was considered significant.

## Results

### Association of *E. coli* Pathotypes with Gastroenteritis

After excluding samples for which patient data were incomplete (12 samples), which were negative by PCR for *lacZ* (45 samples), or which gave equivocal results in the PCR or DNA hybridization assays (8 samples), 1,185 (94.8%) samples of the original 1,250 were available for analysis: 696 from patients with gastroenteritis and 489 from healthy persons. The results of the assays are summarized in Table 4. Enterotoxigenic (ETEC) and enteroinvasive (EIEC) strains of *E. coli* and Bfp-positive EPEC were identified in <0.5% of healthy persons or patients with gastroenteritis. EHEC were present in 4 (0.6%) of 696 of samples from persons with gastroenteritis and in no healthy persons, but the difference between the two groups was not significant (p = 0.15, Fisher exact test, 2-tailed). In contrast, both EAEC and atypical (Bfp-negative) EPEC were identified in >5% of patients, and the difference between the symptomatic and baseline groups was significant for each of these pathotypes (Table 4). Analysis of the data pertaining to patients in whom EAEC and atypical EPEC were identified showed that atypical EPEC were more frequent in younger persons; patients with atypical EPEC were a median age of 3.4 years, compared with 7.4 years for the symptomatic group overall (p < 0.0001; Mann-Whitney test, 2-tailed). Of all atypical EPEC in study participants with gastroenteritis, 75 (84%) were identified in children <10 years old, compared with 14 (16%) in patients >10 years (relative rate [RR] 3.4, 95% confidence interval [CI] 2.0–5.9). In contrast, the median age of patients infected with EAEC was 7.3 years.

**Table 4:** Frequency of *Escherichia coli* pathotypes in study participants with and without gastroenteritis

*E. coli* pathotype^a^	Source		
	Symptomatic (n = 696) (%)	Symptom-free (n = 489) (%)	p^b^
EAEC	45 (6.5)	15 (3.1)	0.02
Typical EPEC	2 (0.3)	1 (0.2)	NS
Atypical EPEC	89 (12.8)	11 (2.3)	< 0.0001
EIEC	0	0	NS
ETEC	2 (0.3)	0	NS
EHEC	4 (0.6)	0	NS
STEC, not EHEC	0	1 (0.2)	NS

^a^EAEC, enteroaggregative *E. coli*; EPEC, enteropathogenic *E. coli*; EIEC, enteroinvasive *E. coli*; ETEC, enterotoxigenic *E. coli*; STEC, Shiga toxin–producing *E. coli*; EHEC, enterohemorrhagic *E. coli*; NS, not significant (p > 0.1). ^b^Fisher exact test, 2-tailed.

**Figure 1:**
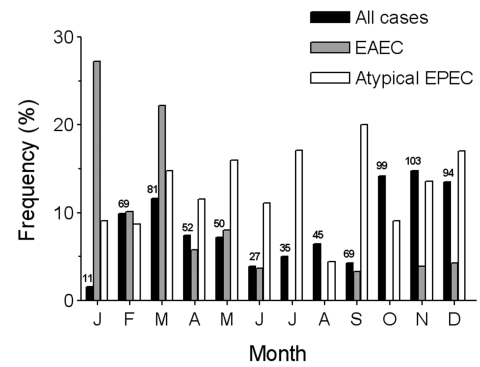
Seasonal incidence in gastroenteritis in the Melbourne Water Quality Study, 1998. Solid black bars indicate all cases of gastroenteritis as a percentage of the total, with the number of cases indicated above each bar. The frequencies of enteroaggregative *Escherichia coli* (EAEC) and enteropathogenic *E. coli* (EPEC) are expressed as a percentage of all cases examined each month.

Examining the seasonal occurrence of gastroenteritis from all causes showed that gastroenteritis was more common in the warmer months; 65.7% of cases occurred in the 6 months from October through March (Figure 1). Infections with EAEC and atypical EPEC reflected this distribution with 80% and 65.5% of infections with these bacteria, respectively, occurring during the same period. The high incidence of EAEC in February and March may have been caused by small family outbreaks; 9 of 25 cases during this period originated in three households.

In view of the seasonal variation in the occurrence of EAEC and atypical EPEC, we reanalyzed the data and compared the symptomatic and baseline groups, because the EPEC were examined only during the 4-month period from May through August, 1997. This analysis showed that the occurrence of EAEC in persons with gastroenteritis (6 [3.8%] of 157) and in asymptomatic persons (15 [3.1%] of 489) was essentially the same during May through August of 1998 and 1997, respectively (RR 1.2, 95% CI 0.5–3.0). In contrast, atypical EPEC were more frequent in the gastroenteritis group during the same period; 19 (12.1%) of 157 of symptomatic patients were positive for these bacteria, compared with 11 (2.3%) of 489 for the asymptomatic group (RR 5.7, 95% CI 3.1–10.5, p < 0.0001; Fisher exact test, 2-tailed). The frequency of atypical EPEC in symptomatic patients from households with real and sham water treatment units was similar (42 [12.3%] of 341 and 47 [13.2%] of 355, respectively).

### Characterization of EPEC Isolates

Pure cultures of PCR- and probe-positive atypical EPEC were obtained from 22 randomly selected samples that were positive in the original PCR. These isolates were characterized further to establish their identity as EPEC and to determine if they belonged to a limited number of clones. All strains were identified as atypical EPEC in that they were negative for *bfpA*, *stx1*, *stx2*, *aggA*, and *aggR*. Determination of O:H serotype and intimin subtype revealed that although strains from each sample were the same, those obtained from different persons were highly heterogeneous, and no two isolates belonged to the same serotype and intimin subtype (Table 5). Only three isolates were of serotypes (O55:H7 and O126:H6 [two isolates]) that commonly include atypical EPEC. In addition, one isolate, W145, was serotype O55:H6, which includes typical EPEC strains (*2*). Eight isolates were O-serogroups that were classified as nontypeable because they did not react with any of the available O-typing sera (O1–O181), and one isolate had an H antigen that did not react with any of the available H-typing sera (H1–H56) and was classified as nontypeable.

**Table 5:** Characteristics of 22 isolates of atypical enteropathogenic *Escherichia coli* identified during this study

Strain	Sou-rce^a^	Serotype	Intimin type	Result of PCR^b^							
				*bfpA*	*aggR*	*efa1*	*astA*	*lpfD*	Adhesion pattern^a^	FAS assay	HEp-2 cell invasion^c^
W7	N	O161:H40	β	–	–	–	–	–	NA	–	0.01
W85-2	N	OR:H-	γ	–	–	–	–	–	AA	+	0.02
W114	N	O107:H8	ι	–	–	–	–	–	IA	+	0.02
W143	N	Ont:H5	ε	–	–	–	–	–	IA	+	< 0.01
W145	N	O55:H6	γ	–	–	+	+	–	AA	+	0.03
W154	N	O139:H14	β	–	–	–	–	–	AA	+	< 0.01
W185	N	Ont:H21	θ	–	–	–	–	–	IA	+	0.02
W208	N	Ont:H4	β	–	–	–	–	–	IA	+	0.08
W761	N	O124:H40	λ	–	–	–	–	–	NA	–	0.07
W902	N	O125:H6	α2	–	–	–	–	–	IA	+	0.06
W914	N	O125:H6	α	–	–	–	–	–	AA	+	0.07
W1040	G	O15:Hnt	β	–	–	+	–	+	LAL	+	0.10
W1056	G	O55:H7	γ	–	–	+	+	–	IA	+	0.77
W1068	G	O51:H49	α	–	–	–	–	–	AA	+	< 0.01
W1082	G	Ont:H6	β	–	–	–	–	–	AA	+	0.01
W1092	G	OR:H-	η/ε	–	–	–	–	+	IA	+	< 0.01
W1108	G	O172:H4	θ	–	–	–	–	–	IA	+	0.03
W1118	G	O126:H6	α	–	–	–	–	–	IA	+	0.01
W1120	G	Ont:H34	α	–	–	–	–	–	IA	+	0.01
W1134	G	Ont:H6	θ	–	–	–	–	–	NA	–	0.01
W1585	G	Ont:H40	θ	–	–	–	–	–	NA	–	0.02
W1706	G	Ont:H6	α	–	–	–	–	–	AA	+	0.01
E128012	C	O114:H2	β	–	–	+	–	+	IA	+	0.32

^a^N, no symptoms; G, gastroenteritis; C, control strain; Ont, O nontypable (O1–O181); Hnt, H nontypable (H1–H56); NA, nonadherent; AA, aggregative adherence pattern; IA, indeterminate adherence pattern; LAL, localized-like adherence pattern; FAS, fluorescent actin staining. ^b^PCR, polymerase chain reaction; +, positive; –, negative. ^c^Data are the number of bacteria recovered from HEp-2 cells after treatment with gentamicin as a percentage of the total number of cell-associated bacteria and are the mean of two separate assays.

Atypical EPEC strains were also heterogeneous in their carriage of putative accessory virulence determinants (Table 5). Although all bacteria were, by definition, negative for *bfpA*, nearly all were also negative for *efa1* and *lpfD*, the genes for factors that have been implicated as adhesins of some attaching-effacing strains of *E. coli* (*19**,**25*). Only two strains were positive for *astA*, which has also been suggested to contribute to the virulence of atypical EPEC (*26*).

### Adhesion to HEp-2 Cells

**Figure 2:**
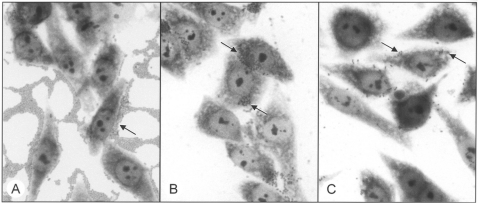
Patterns of adherence of atypical enteropathogenic *Escherichia coli* strains (arrows) to HEp-2 epithelial cells. A) aggregative adherence, B) localized-like adherence, and C) indeterminate adherence. Magnification x1,000.

**Figure 3:**
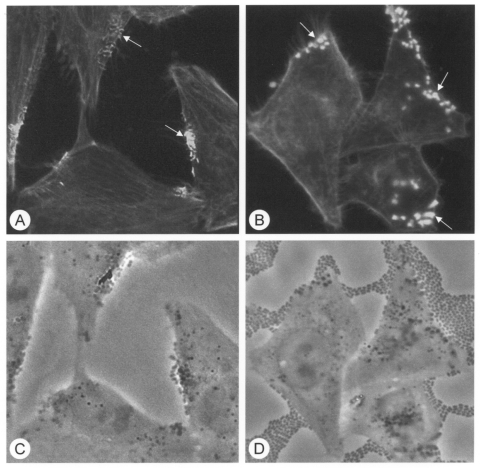
Fluorescent actin staining (FAS) assay for attaching-effacing capacity of atypical enteropathogenic *Escherichia coli* (EPEC) strains with different patterns of adherence to HEp-2 cells. Fluorescent micrographs of HEp-2 cells (A and B) incubated with strains of atypical EPEC showing localized-like and aggregative adherence, respectively, and then reacted with fluorescein-labelled phalloidin. Note the foci of intense fluorescence (arrows) associated with adherent bacteria, which were also visualized by phase contrast microscopy of the same microscope fields (C and D). Magnification x1,000.

Typical EPEC adhere to HEp-2 cells in a distinctive pattern termed localized adherence, which requires the presence of Bfp (*5*). The 22 atypical EPEC strains isolated in this study showed variable patterns of adherence to HEp-2 cells, including aggregative adherence (7 strains) and a pattern previously termed localized-like adherence (1 strain) (*27*). Ten strains showed an indeterminate pattern of adherence, with small numbers of bacteria distributed apparently at random on the cell surface (Figure 2), and 4 strains were classified as nonadherent. The frequency of strains displaying each pattern of adherence was similar among isolates from patients with gastroenteritis and healthy persons. All atypical EPEC strains that adhered to HEp-2 cells, regardless of their pattern of adhesion, were positive in the FAS assay (Figure 3), which indicates that the LEE in these bacteria is functional. All nonadherent strains hybridized with DNA probes were derived from different regions of LEE, which suggests that they carry the entire pathogenicity island. We did not attempt to determine whether LEE was functional in these bacteria.

Previous reports of atypical EPEC have suggested that invasion of epithelial cells may contribute to the virulence of these bacteria (*28*). In this study, however, only one strain, W1056 (O55:H7), was able to invade HEp-2 cells to a noticeable extent (Table 5).

## Discussion

The role of EPEC as a cause of diarrhea in children, particularly in developing countries, is now well established (*2**,**3*). The proven virulence determinants of EPEC include genes within the LEE, notably intimin (the outer membrane protein product of the *eae* gene), and Bfp, which is encoded by EAF (*5*). The key role of EAF in promoting the virulence of EPEC was established by Levine et al. (*12*), who showed that an EAF-negative derivative strain of EPEC, E2348/69, is markedly less virulent for adult volunteers than the wild-type strain. The same study showed that an atypical EPEC strain, E128012, which intrinsically lacks EAF, is also virulent in volunteers. This observation established that certain EPEC strains do not require EAF to cause disease. These intrinsically EAF-negative strains were originally called Class II EPEC (*2**,**3*) but are now more generally referred to as atypical EPEC. They are characterized by the presence of LEE and the absence of factors encoded by EAF, in particular, Bfp. In this way, atypical EPEC resemble EHEC, which are able to cause diarrhea despite their lack of Bfp. Indeed, persuasive evidence indicates that the most prevalent EHEC strain, serotype O157:H7, evolved from an atypical EPEC strain of serotype O55:H7 (*29*).

The aims of the WQS were to investigate the effect of household water treatment units on the incidence of gastroenteritis in Melbourne and to identify causative agents of gastroenteritis in the study population. The microbiologic investigations showed that the detection rate of EAEC (6.5%) and atypical EPEC (12.3%) in patients with diarrhea was greater than that of *Campylobacter* spp. (3%), *Salmonella* spp. (1.1%), adenovirus (1.1%), rotavirus (1.4%), *Cryptosporidium* spp. (1.6%), and *Giardia* spp. (2.5%) and was matched only by that of noroviruses (11.4%) (*6**,**7**,**30*).

Together, atypical EPEC and EAEC accounted for 19.3% of all cases; 21% of cases were attributable to all other bacterial, viral, and parasitic causes combined. However, the frequency of EAEC in patients with gastroenteritis and that in the baseline group without diarrhea was the same when matched for the time of year when the sample of feces was collected. In contrast, atypical EPEC was isolated significantly more often from patients with gastroenteritis than from those without symptoms, regardless of when the sample was collected. A subset of 22 randomly selected atypical EPEC strains was examined and found to be highly heterogeneous, which indicates that the high frequency of atypical EPEC in the study population was not the result of one or more outbreaks attributable to a small number of strains.

Despite the persuasive evidence of a volunteer study and reports of outbreaks of diarrhea with atypical EPEC (*12**,**26**,**31*), the role of atypical EPEC in disease is controversial. Originally, atypical EPEC were grouped with EPEC but were then segregated because they lack EAF. Justification for this division stemmed from the observation that EAF-bearing EPEC far outnumber atypical EPEC as the cause of infantile diarrhea in less-developed countries and of diarrhea outbreaks in general (*3*). In recent reports, however, from countries as diverse as Iran, Poland, South Africa, and the United Kingdom, atypical EPEC strains have outnumbered typical strains as a cause of gastroenteritis (*32**–**35*). Atypical EPEC were also more frequent than typical strains in aboriginal children hospitalized for diarrhea in the Northern Territory of Australia (*36*). These findings were reflected in the present study: 89 (94%) of *eae*-bearing strains identified in patients with gastroenteritis were atypical EPEC.

As for EPEC in general, atypical EPEC were originally incriminated as intestinal pathogens by virtue of their epidemiologic association with cases of diarrhea (*2*). Subsequently, these strains, which had been identified by serotype alone, were shown to be EPEC sensu stricto (*37*). Although atypical EPEC generally are serotypes which differ from EAF-positive EPEC (and other pathotypes of diarrheagenic *E. coli*), the 12 O-serogroups recognized by the World Health Organization as EPEC (i.e., serogroups O26, O55, O86, O111, O114, O119, O125, O126, O127, O128, O142, and O158) include both typical and atypical varieties (*3*). Some of the atypical EPEC strains within these serogroups carry accessory virulence-associated determinants such as the EHEC hemolysin (commonly found in serotypes O26:H11 and O111ac:H8). Some strains also carry *astA*, the gene for enteroaggregative heat-stable enterotoxin, EAST1, which is frequently found in serotypes O55:H7, O119:H2, and O128:H2 (*3*). Atypical EPEC strains of non-EPEC serogroups generally do not express these factors. In the present study, none of the 89 atypical strains was positive for *ehxA*, which is required for the production of EHEC hemolysin, and although two strains (both serogroup O55) tested positive for *astA*, both carried the previously described mutations in this gene, which would preclude the synthesis of biologically active enterotoxin (*38*).

Unlike EAF-bearing strains of EPEC, which show localized adherence to HEp-2 cells, atypical EPEC show different patterns of adherence. Although some investigators have reported localized-like adherence as a predominant adherence pattern of atypical EPEC (*28**,**39*), only 1 of 22 strains investigated here displayed this phenotype. The low frequency of localized-like adherence in this study may reflect differences in serotype distribution, as localized-like adherence seems to be most prevalent in atypical EPEC strains in EPEC serogroups, such as O26, O111, and O119 (*28*), which were infrequently found in this study. Ten of the 22 strains examined exhibited an adherence pattern that was distinct from localized, aggregative, or diffuse adherence (*3*) and was termed indeterminate adherence (Figure 1). This pattern may have been termed diffuse adherence by other investigators, which would account for the relatively high frequency of diffusely adherent atypical EPEC in some reports and their absence from this study. Seven of 22 strains displayed aggregative adherence despite their lack of known sequences associated with the production of fimbriae of EAEC.

The virulence of atypical EPEC despite their lack of Bfp, which typical EPEC require to cause severe disease, suggests that these bacteria carry an adhesin analogous to Bfp that augments their ability to colonize the intestine. Previous studies, however, have shown only a low frequency of known *E. coli* adhesins, including aggregative adherence fimbriae, P fimbriae, S fimbriae, PAP pili, and afimbrial adhesins in atypical EPEC strains (*40*). We extended these findings to show that atypical EPEC are also mostly negative for Efa1 and long polar fimbriae (encoded by *lpfD*), which contribute to the adhesive capacities of some attaching-effacing strains of *E. coli* (*19**,**25*). Although atypical EPEC may carry an adhesin equivalent to Bfp, which remains to be discovered, these bacteria may use any known *E. coli* adhesins to bind to the intestine. This suggestion is supported by the marked heterogeneity of atypical EPEC in terms of adhesion pattern and the observation that adhesins other than Bfp can restore cell-binding capacity to EAF-cured strains of typical EPEC (*41*).

In conclusion, atypical EPEC were the most commonly identified pathogens in a study of community-acquired diarrhea in Melbourne. Infections with atypical EPEC occurred throughout the year and were significantly more common in children. Characterization of a sample of atypical EPEC isolates revealed that these bacteria were antigenically heterogeneous and generally did not belong to O-serogroups associated with EPEC. Further studies are needed to determine the frequency of these bacteria in other communities, their reservoir, mode of spread, and mechanisms of virulence.
